# Pregnant women lose all hope of help as Pakistan drowns in floods

**DOI:** 10.1002/hsr2.1082

**Published:** 2023-10-11

**Authors:** Aiman Idress, Aqeeb Ur Rehman, Aleenah Mohsin, Ka Yiu Lee

**Affiliations:** ^1^ Department of Community Medicine King Edward Medical University Lahore Pakistan; ^2^ Department of Health Sciences Mid Sweden University Sweden

Dear Editor,

The massive monsoon flooding in Pakistan has drowned nearly one‐third of the country and has baffled both the government and the medical communities. The magnitude of devastation is expected to have surpassed even that of the 2010 floods. While social issues like population displacement, infrastructural damage, infectious diseases, unemployment and decreased crop production draw immediate attention, other more chronic issues that linger for a longer time after the floods, have not received satisfactory attention. One of the most important issues is obstetrics‐related care for pregnant women. According to a report, at least 650,000 women in flood‐affected areas in Pakistan are pregnant, with about 73,000 of them expecting delivery within a month,^1^ warranting it to be a public health emergency. However this issue has not received much media and government attention. There is an urgent need to cater to the medical and obstetric needs of these women and newborns as an absolute and inseparable part of flood relief policies.

Mother‐Child care is a subject that has been receiving inadequate attention in Pakistan even before the floods. Pregnancy outcomes in Pakistan are much worse than those in other lower middle‐income countries (LMICs) including India, Congo, Guatemala, Kenya, and Zambia. Most women in reproductive ages are uneducated and undernourished, leading to maternal and neonatal mortalities being more than double than in the aforementioned LMICs.^2^ Flood has only aggravated an already adverse situation for the most vulnerable and oft‐neglected communities in Pakistan. Considering the lack of hospital access and maternity care available for pregnant women in flood‐affected areas, the indicators for mother‐child health are expected to worsen even further.^3^ Studies of flood‐stricken areas in the past have shown that rates of low birth weight and gestational hypertension increase exponentially after calamities of magnitude such as this one.^4^ These complications may even require hospitalization, lifesaving medicines and procedures, which are the most basic necessities but have become luxuries in the worst affected areas in Pakistan. Increased emotional and financial stressors along with the added physical distress of infectious diseases^5^ are the unavoidable constants in these areas that have further added to the poor prenatal, natal, and antenatal health conditions of mothers and their children. Furthermore, as expected in flood‐affected areas, food, and medication are insufficient to meet the needs of the people in general, let alone the pregnant women. Not to forget the COVID‐19 pandemic, which is also expected to surge in the background of overcrowding in relief camps. All these factors have put pregnant women and their children at a risk greater than the general population. Unless substantial measures are taken in this regard, consequences can be permanent and fatal for a large proportion of the population. Thus, immediate intervention on obstetric care on part of the government as well as the medical fraternity is an absolute need of the hour.

Ensuring “clean birth” in flood‐affected areas is a huge challenge owing to a lack of aseptic conditions, leaving women and children increasingly vulnerable to sepsis and infections. ‘Maternity kits’ comprising of gloves, plastic sheets for a dry birthing surface, clean blades, thread and swabs for the umbilical cord, baby clothes, soap, and multivitamins have been introduced. This initiative must be extended by the government and NGOs on an extensive scale, making these kits available for as many pregnant women as possible in the flood‐affected areas. We recommend that the authorities concerned must pre‐emptively lay standard operating procedures and take measures to ensure that prenatal, antenatal, and postnatal care are adequate for pregnant women in areas that are at risk of flooding. This includes special heed to the provision of hospital access and deployment of lady healthcare workers, midwives, doctors and trained birth attendants for services in these areas in case of flooding in the monsoon. This will help reduce complications to a minimum and ensure adequate prenatal care and clean deliveries. Moreover, pregnancies at risk for complications must be identified so that women can be moved to hospitals over predesignated safe routes in case of expected complications. Above all, attention must be paid to hygiene and sanitation in the flood‐affected areas for the control of infectious diseases, hence improving maternal and neonatal outcomes.

## AUTHOR CONTRIBUTIONS

**Aiman Idrees**: Conceptualization, supervision, writing—original draft. **Aqeeb Ur Rehman**: Data curation, validation, writing—review & editing. **Aleenah Mohsin**: Conceptualization, data curation, writing—original draft. **Ka Yiu Lee**: Supervision, validation, writing—review & editing.

## CONFLICTS OF INTEREST STATEMENT

The authors declare no conflict of interest.

## TRANSPARENCY STATEMENT

The lead author and guarantor affirms that this manuscript is an honest, accurate, and transparent account of the study being reported and that no important aspects of the study have been omitted.

## Data Availability

Data sharing is not applicable to this article.
